# Association between cumulative cigarette and Waterpipe smoking and symptoms of dependence in Lebanese adults

**DOI:** 10.1186/s12889-021-11626-7

**Published:** 2021-08-23

**Authors:** Diana Malaeb, Marwan Akel, Hala Sacre, Chadia Haddad, Sahar Obeid, Souheil Hallit, Pascale Salameh

**Affiliations:** 1 School of Pharmacy, Lebanese International University, Beirut, Lebanon; 2 INSPECT-LB (Institut National de Santé Publique, d’Épidémiologie Clinique et de Toxicologie-Liban), Beirut, Lebanon; 3 Univ. Limoges, UMR 1094, Neuroépidémiologie Tropicale, Institut d’Epidémiologie et de Neurologie Tropicale, GEIST, 87000 Limoges, France; 4 Faculty of Arts and Sciences, Holy Spirit University of Kaslik (USEK), Jounieh, Lebanon; 5 Faculty of Medicine and Medical Sciences, Holy Spirit University of Kaslik (USEK), Jounieh, Lebanon; 6 Faculty of Pharmacy, Lebanese University, Hadat, Lebanon; 7 University of Nicosia Medical School, Nicosia, Cyprus

**Keywords:** Cigarette, Waterpipe, Dependence, Symptoms, Cumulative

## Abstract

**Background:**

Waterpipe and cigarette smoking dependence are becoming increasingly prevalent forms of addiction globally. This study examined whether cumulative cigarette smoking and cumulative waterpipe smoking are associated with higher dependence on both cigarettes and waterpipe.

**Methods:**

This cross-sectional study conducted between February and April 2020, enrolled 363 participants drawn from all Lebanese districts. The mean age was 29.51 years, 64.8% were females, and 124 (34.2%) exclusive cigarette smokers, 189 (52.1%) exclusive waterpipe smokers, and 50 (13.8%) dual smokers (waterpipe and cigarette). We used the Hooked on Nicotine Checklist (HONC) as an indicator of decreased autonomy towards nicotine, in addition to the Lebanon Waterpipe Dependence Scale-11 (LWDS11) and the Lebanese Cigarette Dependence scale (LCD). A stepwise linear regression was performed taking the HONC scores due to cigarette and waterpipe smoking, LCD and LWDS-11 scores as dependent variables.

**Results:**

The results showed that in the total sample, higher cumulative cigarette smoking (B = 0.005 with a confidence interval of 0.004, 0.006) was significantly associated with higher HONC cigarette scores, whereas higher cumulative waterpipe smoking (B = -0.006 with a confidence interval of − 0.009, − 0.002) was significantly associated with lower HONC cigarette scores. Moreover, higher cumulative waterpipe smoking (B = 0.012 with a confidence interval of 0.009,0.015) was significantly associated with higher HONC waterpipe scores. The results showed that, in both sexes, higher cumulative cigarette smoking was associated with higher HONC cigarette scores and lower HONC waterpipe scores. Furthermore, higher cumulative waterpipe smoking was significantly associated with higher HONC waterpipe scores in both sexes.

**Conclusion:**

Our study supports the fact that heavy nicotine consumption, related to both the increased frequency and smoking duration, can increase the risk of dependence. It raises the need for strategic plans to minimize and discourage the use of nicotine products in Lebanese community settings.

**Supplementary Information:**

The online version contains supplementary material available at 10.1186/s12889-021-11626-7.

## Background

Cigarette use is the most prevalent form of tobacco consumption worldwide [1]. Nevertheless, waterpipe tobacco smoking, one of the alternative forms of tobacco use, has steadily gained popularity [2–4]. The increase in waterpipe tobacco smoking and other non-cigarette tobacco products in several Eastern Mediterranean and Eastern European countries has led to an increased prevalence of dual and poly-tobacco use, raising public health concerns in these countries [5]. In particular, Lebanon’s most recent measures (in 2020) of tobacco smoking prevalence were high among adults: 45.2% for cigarettes [6], 36.9% for waterpipe [7], and 4.2% for dual smoking (above 40 years; in 2011) [8]. Dual cigarette and waterpipe smoking hold a high risk of dependence explained by the combined ease of carrying a pack of cigarettes in one’s pocket, with the socially pleasant opportunity to use waterpipe tobacco smoking when it is available [9].

Dual smokers are more dependent on waterpipe smoking due to the perception that it is less harmful than cigarettes [10] and an overarching high social dependence that complements the neuropharmacological changes, confirming it as an established smoking modality favoring dependence [11–13]. Additionally, dual smokers face roadblocks and barriers associated with lower interest in quitting [14]. As waterpipe smoking exerts a social dependence and a pleasurable experience, cigarette smoking fulfills the needs of individuals and can be viewed as a coping strategy for stress and nicotine cravings [15]. A study conducted in 17 Arab countries showed a prevalence of 3.8% dual smokers, higher among males [16]. Modeling estimates suggest that waterpipe tobacco users who also use cigarettes smoke both products more frequently and intensely than those who only use one smoking method [17].

Dependence is defined as the reduced autonomy over the use of tobacco products, added to the development of specific symptoms resulting from behaviors relating to the use of these products [18]. Back in 2002, DiFranza et al. [19] had described autonomy loss as the sequelae of tobacco use, either physical or psychological, presenting a barrier to quitting. Some manifestations of dependence include cravings, physical addiction, withdrawal symptoms during periods of abstinence, loss of control over the amount or duration of use, and tolerance symptoms, all predictive hallmarks of the risk for future smoking behavior and nicotine dependence [20, 21, 22].

Various scales assess nicotine dependence, such as the Hooked on Nicotine Checklist (HONC) that measures the degree of autonomy loss induced by tobacco products [19, 23, 24]. Other scales generated in the Lebanese adult population measure the two most common types of tobacco dependence, i.e., the Lebanese Cigarette Dependence scale (LCD) [23] and Young Adults Cigarette Dependence (YACD) [25], inspired by the Fagerström Test for Nicotine Dependence and its revision [26], and the Waterpipe Dependence Scale (LWDS-11) [24].

However, very few studies used both tools in the same population, and none has ever assessed the comparative effect of cumulative tobacco smoking on dependence. Hence, the study objective is to assess whether cumulative cigarette smoking and cumulative waterpipe smoking are associated with higher dependence on both cigarettes and waterpipe.

## Methods

### General study design

A cross-sectional study carried out between February and April 2020, during the lockdown period imposed by the government for the COVID-19 pandemic, enrolled a convenient sample of 363 community-dwelling participants using the snowball sampling technique. Due to the restrictions on gatherings, the non-practical and risky side of face-to-face interviews, the survey was created on Google forms (https://bit.ly/3m80yls) and made available on social media (WhatsApp, LinkedIn, and Facebook); participants were asked to share the link with other smokers. Participation was voluntary, and participants had the right to withdraw at any time by not completing the questionnaire, which was anonymous and respected the confidentiality of participants. All cigarette or waterpipe smokers above 18 were eligible to participate by answering the question: “Are you a current smoker?”; current smoker status was clarified as smoking at least one waterpipe or cigarette in the last 30 days. Participants who did not meet one of these two definitions were considered non-smokers. Cumulative waterpipe smoking and cumulative cigarette smoking were calculated by multiplying the number of waterpipes smoked per week and the number of cigarettes smoked per day by the number of years of smoking.

### Sample size calculation

The G-power software calculated a minimum sample of 315 participants, based on a minimum change of 5% in the R^2^, a confidence level of 95%, a power of 80%, and ten predictors to be entered in the final model of multiple regressions. This sample size was then increased by 15% to account for possible missing or illogical values within the database.

### Questionnaire and variables

The self-administered questionnaire (Appendix 1) was in Arabic (the native language in Lebanon), with closed-ended questions, and required approximately 15–20 min to complete. It collected information about the socio-demographic characteristics: age, sex, district, marital status, work status, education level (divided into primary, intermediate, secondary, and university), and the household crowding index, calculated by dividing the number of persons living in the house by the number of rooms, excluding the kitchen and bathrooms.

The second and third sections were intended for cigarettes and waterpipe smokers, respectively, and consisted of the following scales used to assess nicotine dependence:

1. The Hooked on Nicotine Checklist (HONC) consists of ten items and serves as an indicator of decreased autonomy. A score of zero obtained by answering No to all ten questions indicates full autonomy over tobacco use [19]. The scale was applied twice in dual smokers: once for cigarette smoking and once for waterpipe smoking; thus, these participants had two measures for the HONC scale (HONC Cigarettes Cronbach’s alpha = 0.868 and HONC Waterpipe Cronbach’s alpha = 0.853).
2. The Lebanon Waterpipe Dependence Scale-11 (LWDS11) is used to assess waterpipe dependence [24]. It includes 11 items rated on a 4-point Likert scale from 0 to 3. The total score is calculated by summing the 11 responses. (Cronbach’s alpha = 0.774).
3. The Lebanese Cigarette Dependence scale (LCD) is a comprehensive tool used to assess cigarette smoking dependence [23]. It includes 20 items measured on a 4-point Likert scale. The total score is calculated by summing all answers. (Cronbach’s alpha = 0.828).
4. This study did not use the Diagnostic and Statistical Manual of Mental Disorders (DSM-5) or the International Classification of Diseases-10 (ICD-10) because both the LWDS-11 and LCD are validated in the Lebanese population, while DSM-5 evaluates withdrawal and tolerance but not dependence [27].

### Statistical analysis

The statistical analysis was performed using SPSS software, version 23. The normality of distribution of the HONC, LCD, and LWDS scores was confirmed via a calculation of the skewness and kurtosis; values for asymmetry and kurtosis between − 2 and + 2 are considered acceptable to demonstrate normal univariate distribution [28]. These conditions consolidate the assumptions of normality in sample sizes larger than 300 [29]. The Student t-test was used to check for an association between the LCD, LWDS-11, and cigarette and waterpipe HONC scores and dichotomous variables, while the ANOVA test was used to compare the means of three or more groups. Pearson correlation was used to compare two continuous variables.

Finally, four stepwise linear regressions were conducted, taking the HONC scores due to cigarette and waterpipe smoking, LCD and LWDS-11 scores as outcomes; to minimize confounding, independent variables entered in the final model were those that showed a *p* < 0.2 in the bivariate analysis. Assumptions of linear regressions were checked and were assumed to be acceptable before accepting the final models. Moreover, stratified analyses over sex and age groups were conducted. In all cases, a value of *p* < 0.05 was considered significant.

## Results

In our sample (*N* = 363), 64.8% were females, and the mean age was 29.51 years [95% CI 28.28, 30.73]. The majority had a university level of education and were from Mount Lebanon. Table 1 summarizes all sociodemographic characteristics.

**Table 1:** Sociodemographic characteristics of the participants (N=363)

	Frequency	Percentage (%)
**Sex**		
Male	164	45.2%
Female	199	54.8%
**Marital status**		
Single	239	65.8%
Married	124	34.2%
**Education level**		
Primary and Complementary (10 years or less)	22	6.1%
Secondary (11–13 years)	43	11.8%
University (14 years or more)	298	82.1%
**Employment status**		
Unemployed	160	44.1%
Employed	203	55.9%
**District**		
Beirut	87	24.0%
Mount Lebanon	151	41.6%
North	46	12.7%
South	45	12.4%
Bekaa	34	9.4%
	**Mean**	**95% CI**
**Age (in years)**	29.51	28.28, 30.73
**Household crowding index**	1.00	0.92, 1.08

Participants were divided into three groups: 124 (34.2%) exclusive cigarette smokers, 189 (52.1%) exclusive waterpipe smokers, and 50 (13.8%) dual smokers (waterpipe and cigarette).

The mean age of cigarette smoking initiation was 18.77 years (95% CI 17.46, 20.07), while that of the waterpipe was 18.80 years (95% CI 17.13, 20.47). The mean number of cigarettes smoked per day was 8.32 (95% CI 6.02, 10.63), and the mean number of weekly waterpipes smoked was 3.97 (95% CI 2.91, 5.04). The mean number of years of cigarette and waterpipe smoking was 6.09 (95% 4.99, 7.19) and 5.73 (95% CI 4.96, 6.51), respectively. The cumulative cigarette smoking was 81.86 (95% CI 41.54, 122.19), and that of the waterpipe was 36.84 (95% CI 20.29, 53.39).

### Bivariate analysis

Higher HONC cigarette scores were found in males and those with a primary/complementary level of education. Furthermore, higher HONC cigarette scores were associated with higher cumulative cigarette smoking, older age, and lower cumulative waterpipe smoking.

Higher HONC waterpipe scores were significantly found in females and single participants. Moreover, higher HONC waterpipe scores were associated with higher cumulative waterpipe smoking, less cumulative cigarette smoking, and younger age.

Higher LCD scores were found in males and those with a primary/complementary level of education. Higher LCD scores were associated with higher cumulative cigarette smoking, older age, and lower cumulative waterpipe smoking.

Higher LWDS scores were found in females. Higher LWDS scores were associated with higher cumulative waterpipe smoking, less cumulative cigarette smoking, and younger age (Table 2).

**Table 2:** Bivariate analysis of factors associated with the cigarette and waterpipe dependence scores and symptoms of dependence (HONC scores) due to cigarette and waterpipe smoking (*N* = 363)

Variable	HONC cigarette	HONC waterpipe	LCD score	LWDS score
**Sex**				
Male	3.23	1.18	9.70	4.89
Female	1.60	2.52	4.56	7.73
*p*	**< 0.001**	**< 0.001**	**< 0.001**	**< 0.001**
95% CI of the mean difference	0.96, 2.30	−1.86, −0.83	3.28, 6.98	−4.15, − 1.53
**Marital status**				
Single/divorced/widowed	2.18	2.13	6.26	6.84
Married	2.62	1.48	8.09	5.69
*p*	0.253	**0.021**	0.08	0.113
95% CI of the mean difference	−1.19, 0.32	0.10, 1.20	−3.89, 0.22	−0.27, 2.56
**Education level** means (95% CI)				
Primary and Complementary (10 years or less)	4.91 (3.01, 6.80)	2.55 (1.09, 4.00)	12.27 (7.17, 17.37)	8.73 (5.06, 12.40)
Secondary (11–13 years)	3.37 (2.27, 4,48)	1.91 (0.95, 2.87)	9.44 (6.47, 12.41)	6.86 (4.54, 9.18)
University (14 years or more)	1.99 (1.65, 2.34)	1.87 (1.58, 2.15)	6.11 (5.14, 7.09)	6.22 (5.51, 6.92)
*p*	**0.001**	0.485	**0.005**	0.369
**District** means (95% CI)				
Beirut	2.32 (1.62, 3.02)	2.16 (1.62, 2.70)	6.48 (4.49, 8.47)	7.24 (5.74, 8.74)
Mount Lebanon	2.22 (1.70, 2.73)	1.45 (1.09, 1.81)	6.60 (5.19, 8.01)	5.37 (4.53, 6.21)
North	3.11 (2.02, 4.20)	2.50 (1.57, 3.43)	9.20 (6.40, 11.99)	7.80 (5.54, 10.07)
South	1.80 (0.88, 2.72)	1.89 (1.05, 2.72)	6.13 (3.45, 8.81)	6.60 (4.37, 8.83)
Bekaa	2.53 (1.35, 3.70)	2.56 (1.40, 3.72)	7.00 (3.78, 10.22)	7.15 (4.70, 9.60)
*p*	0.401	0.116	0.459	0.429
**Cumulative cigarette smoking** r (95% CI)	0.420^a^ (0.336, 0.504)	−0.215^a^ (− 0.307, − 0.124)	0.515^a^ (0.439, 0.591)	− 0.265^a^ (− 0.347, − 0.184)
**Cumulative waterpipe smoking** r (95% CI)	−0.188^a^ (− 0.282, − 0.095)	0.333^a^ (0.244, 0.422)	−0.205^a^ (− 0.297, − 0.113)	0.414^a^ (0.338, 0.489)
**Age** r (95% CI)	0.180^b^ (0.051, 0.309)	− 0.159^c^ (− 0.289, − 0.03)	0.259^a^ (0.133, 0.385)	− 0.152^c^ (− 0.270, − 0.034)
**Household crowding index** r (95% CI)	0.095 (− 0.005, 0.195)	−0.028 (− 0.128, 0.072)	0.051 (− 0.048, 0.150)	−0.03 (− 0.121, 0.062)

^a^*p* < 0.001; ^b^ *p* < 0.01; ^c^ *p* < 0.05; numbers in bold indicate significant *p*-values; CI=Confidence Interval, numbers are presented as means (95% CI) or rho (95% CI). HONC=Hooked on Nicotine Checklist; LWDS = Lebanon Waterpipe Dependence Scale; LCD = Lebanese Cigarette Dependence scale

### Dual versus exclusive smokers

When assessing whether dual users had higher cumulative use scores than either exclusive cigarette or exclusive waterpipe smokers, the results showed that dual smokers significantly had lower cumulative cigarette smoking than exclusive cigarette smokers (*p* < 0.001), whereas no significant difference was seen between exclusive waterpipe smokers and dual smokers in terms of cumulative waterpipe smoking (Table 3). When stratified over median age (=25), these results were maintained among participants aged 25 years and above, while they were inversed among younger people (< 25 years): dual young smokers smoked similar amounts of cigarettes than exclusive cigarette smokers, but lower quantities of waterpipe than exclusive waterpipe smokers (Table 3).

**Table 3:** Association between smoking status, cumulative cigarette and waterpipe smoking

Whole sample (***N*** = 363)		
Smoking status	Cumulative cigarette smoking	Cumulative waterpipe smoking
Exclusive cigarette	321.56 (237.38, 405.75)	–
Exclusive waterpipe	–	54.70 (40.59, 68.81)
Dual smokers	77.82 (31.94,123.69)	52.14 (24.25, 80.02)
*p-value*	p < 0.001	*p* < 0.870
**Among young participants (age < 25 years) (N = 164)**		
**Smoking status**	**Cumulative cigarette smoking**	**Cumulative waterpipe smoking**
Exclusive cigarette	50.30 (29.34, 71.26)	–
Exclusive waterpipe	–	32.32 (21.44, 43.21)
Dual smokers	31.61 (17.35, 45.86)	17.56 (8.14, 26.99)
*p-value*	*P* = 0.139	*P* = 0.042
**Among older participants (age 25 years and above) (*N*** **= 198)**		
**Smoking status**	**Cumulative cigarette smoking**	**Cumulative waterpipe smoking**
Exclusive cigarette	471.39 (353.07, 589.71)	–
Exclusive waterpipe	–	76.38 (51.18, 101.58)
Dual smokers	136.64 (34.96, 238.32)	96.14 (36.51, 155.76)
*p-value*	P < 0.001	*P* = 0.509

Number between parentheses refer to 95% CI

### Multivariable analysis

In the total sample, higher cumulative cigarette smoking (B = 0.005) was significantly associated with higher HONC cigarette scores, whereas higher cumulative waterpipe smoking (B = − 0.006) was significantly associated with lower HONC cigarette scores (Table 4, Model 1).

**Table 4:** Linear regressions using the ENTER method conducted on the whole sample

Model 1 taking the HONC due to cigarette score as the dependent variable.															
Variable	Total sample (N = 363)			Males (N = 164)			Females (***N*** = 199)			Age < 25 years (***N*** = 164)			Age ≥ 25 years (***N*** = 198)		
	**B**	**β**	**95% CI SB**	**B**	**β**	**95% CI SB**	**B**	**β**	**95% CI SB**	**B**	**β**	**95% CI SB**	**B**	**β**	**95% CI SB**
Cumulative cigarette smoking	0.005	0.471	0.004, 0.006	0.005	0.525	0.003, 0.006	0.005	0.419	0.003, 0.007	0.042	0.608	0.034, 0.050	0.005	0.551	0.003, 0.006
Cumulative waterpipe smoking	-0.006	−0.143	− 0.009, − 0.002	− 0.01	− 0.205	− 0.018, − 0.003	− 0.005	− 0.158	− 0.009, − 0.001	− 0.005	− 0.073	− 0.014, 0.004	− 0.005	− 0.159	− 0.009, − 0.001
**Model 2 taking the HONC due to waterpipe score as the dependent variable.**															
**Variable**	**B**	**β**	**95% CI SB**	**B**	**β**	**95% CI SB**	**B**	**β**	**95% CI SB**	**B**	**β**	**95% CI SB**	**B**	**β**	**95% CI SB**
Cumulative cigarette smoking	−0.001	−0.087	−0.002, 0.001	−0.001	− 0.116	−0.002, 0.001	− 0.001	−0.098	− 0.003, 0.001	−0.014	− 0.233	−0.022, − 0.006	−0.001	− 0.182	−0.002, − 0.0001
Cumulative waterpipe smoking	0.012	0.375	0.009, 0.015	0.013	0.419	0.008, 0.018	0.011	0.367	0.007, 0.015	0.031	0.499	0.022, 0.039	0.009	0.369	0.006, 0.013

Moreover, higher cumulative waterpipe smoking (B = 0.012) was significantly associated with higher HONC waterpipe scores, while higher cumulative cigarettes were associated with a lower HONC waterpipe score, although results reached statistical significance only after stratification based on age (Table 4, Model 2).

Indeed, when analyzing the data according to age categories divided according to the median (=25), the results showed that higher cumulative cigarette smoking was significantly associated with higher HONC cigarette scores in participants aged < 25 years and those aged ≥25 years, whereas higher cumulative waterpipe smoking was significantly associated with lower HONC cigarette scores only among those aged ≥25 years. Finally, higher cumulative cigarette smoking was significantly associated with lower HONC waterpipe scores, whereas higher cumulative waterpipe smoking was significantly associated with higher HONC waterpipe scores in those aged < 25 years and those aged ≥25 years (Table 4).

The results showed that, in both sexes, higher cumulative cigarette smoking was associated with higher HONC cigarette scores and lower HONC waterpipe scores. Furthermore, higher cumulative waterpipe smoking was significantly associated with higher HONC waterpipe scores in both sexes (Table 4).

Moreover, when comparing results between strata, no qualitative interaction was visible according to age or sex for both types of smoking, except for the higher cumulative waterpipe associated with lower HONC cigarette scores only among those aged ≥25 years. However, a quantitative interaction related to age was noted with the model coefficients being several folds higher for young participants: cumulative cigarettes being highly associated with HONC cigarettes and HONC waterpipe, while cumulative waterpipe was more associated with HONC waterpipe. As for sex stratification, results were similar among strata (Table 4).

B = unstandardized Beta; β = Standardized Beta; CI=Confidence Interval; HONC = Hooked on Nicotine Checklist.

Covariates entered in the models: HONC Cigarette score: Education, Cumulative cigarette smoking, Cumulative waterpipe smoking, Sex, Age.

HONC waterpipe smoking: Cumulative cigarette smoking, Cumulative watepripe smoking, marital status, Sex, Age.

Moreover, higher cumulative cigarette smoking was significantly associated with more cigarette dependence, whereas higher cumulative waterpipe smoking was significantly associated with less cigarette dependence (Table 5, Model 1). Finally, higher cumulative waterpipe smoking was significantly associated with more waterpipe dependence, whereas higher cumulative cigarette smoking was significantly associated with less waterpipe dependence (Table 5, Model 2).

**Table 5:** Linear regressions using the ENTER method conducted on the whole sample

Model 1 taking the LCD score as the dependent variable.			
Variable	B	β	95% CI
Cumulative cigarette smoking	0.017	0.599	0.014, 0.020
Cumulative waterpipe smoking	−0.014	− 0.130	− 0.023, − 0.005
**Model 2 taking the LWDS score as the dependent variable.**			
**Variable**	**B**	**β**	**95% CI**
Cumulative cigarette smoking	−0.004	−0.175	− 0.006, − 0.001
Cumulative waterpipe smoking	0.038	0.485	0.031, 0.045

*Reference group; numbers in bold indicate significant p-values; B = unstandardized Beta; β = Standardized Beta; CI=Confidence Interval; LCD = Lebanese Cigarette Dependence Scale; LWDS = Lebanese Waterpipe Dependence Scale

Covariates included in the model: LCD score: Sex, Cumulative cigarette smoking, Cumulative waterpipe smoking, Age, Education

LWDS score: Sex, Cumulative cigarette smoking, Cumulative waterpipe smoking, Age

## Discussion

This study is the first to examine whether both cumulative cigarette and waterpipe smoking would be associated with higher dependence, using multiple measures of nicotine dependence, such as HONC, LWDS, and LCD scales. The results also showed that higher cumulative cigarette smoking was significantly associated with higher HONC cigarette scores, whereas higher cumulative waterpipe smoking was significantly associated with lower HONC cigarette scores only among those aged ≥25 years. Moreover, higher cumulative waterpipe smoking was significantly associated with higher HONC waterpipe scores. Results did not differ between males and females. In the same line, higher cumulative cigarette smoking was significantly associated with more cigarette dependence, whereas higher cumulative waterpipe smoking was significantly associated with less cigarette dependence. In addition, higher cumulative waterpipe smoking was significantly associated with more waterpipe dependence, whereas higher cumulative cigarette smoking was significantly associated with less waterpipe dependence. These results were expected, showing that a higher cumulative exposure of any tobacco type is associated with higher nicotine dependence, whether for waterpipe [30–33] or cigarettes [30, 31, 34–37], since smokers tend to sometimes interchange between these two tobacco products to meet the need for nicotine replacement [32].

In the stratified analysis, when comparing results between strata, no qualitative interaction is visible according to age or sex for both types of smoking, except for the higher cumulative waterpipe associated with lower HONC cigarette scores only among those aged 25 years and above. This shows that waterpipe smoking might compensate for cigarette-related nicotine dependence among older adults, but not among youth. In other words, young people might still smoke waterpipe regardless of their nicotine dependence status; waterpipe smoking might be sought for reasons differing from nicotine dependence among youngsters. Indeed, multiple factors elucidate the increased risk of waterpipe dependence among youth, including the positive perception attributed to smoking such as socializing, calming effect, café atmosphere, and the appealing taste/smell of the smoke, thus encouraging and triggering the excessive use of waterpipe [33].

Interestingly, a quantitative interaction related to age was noted, where cumulative cigarettes were highly associated with HONC cigarettes and HONC waterpipe, while cumulative waterpipe was highly associated with HONC waterpipe among younger participants. This result highlights the vulnerability of youth to nicotine dependence compared with older individuals; with similar cumulative exposure to any tobacco type, young adults show higher dependence on nicotine [38].

Our study also showed that dual smokers had significantly lower cumulative cigarette smoking than exclusive cigarette smokers, whereas no significant difference was seen between exclusive waterpipe smokers and dual smokers in terms of cumulative waterpipe smoking. This finding suggests that dual smokers and exclusive cigarettes smokers are heavier smokers than exclusive waterpipe smokers; however, this was only true for adults aged more 25 years and above. Another interpretation could be that cigarettes combine the convenience of smoking, literally at the smoker’s fingertips, to reach a fast kick of nicotine when needed, in addition to the ease and practicality of use [39]. Cigarettes are more accessible than waterpipes, the latter being mainly available at cafes and bars, sometimes considered expensive venues for regular waterpipe use [11], or not frequently visited by older adults. Additionally, nicotine concentration delivered by cigarettes is higher than that of waterpipes, explaining the higher risk of dependence due to cigarettes among smokers [40]. Conversely, the higher cumulative waterpipe smoking is associated with lower HONC cigarette scores which can be mainly explained by the social and friendly setting expected at every waterpipe session [11], an aspect that is attractive for young smokers. Furthermore, waterpipe is considered a form of social smoking in which the pipes are shared among friends and family at home or in bars that favor increased smoking of waterpipe [41].

Our study showed that higher cumulative cigarette smoking was significantly associated with higher HONC cigarette scores in adults < 25 years, consistent with previous findings [42]. Young Lebanese heavily use inexpensive means of smoking, such as cigarettes, which are very cheap in Lebanon [43]. Our results showed that higher cumulative waterpipe smoking was significantly associated with higher HONC waterpipe scores which might be explained by the fact that the waterpipe is more trendy among young adults in Lebanon [43] due to the perception of “no/less harm” of waterpipe, social acceptance accompanied by less restrictions, accessibility, and need for amusement [12].

### Study implications

Our study supports the fact that heavy nicotine consumption, related to both the increased frequency and smoking duration, can increase the risk of dependence. It raises the need for strategic plans to minimize and discourage the use of nicotine products in Lebanese community settings. Our findings show the need for the development of efficient health promotion programs oriented towards the prevention and cessation of smoking practices in the Lebanese population, young people in particular. Furthermore, this study highlights the need to apply policies to reduce smoking through banning tobacco advertising, raising the taxes on tobacco, and adopting measures to curb smoking in public places. The current findings thus stress the need of addition of anti-smoking messages in media channels and increase the price of smoking products to minimize the easy accessibility of such products by young individuals. In addition, Lebanon should promote free consultation and treatment for smokers to remove the roadblocks associated with smoking cessation.

### Strengths and limitations

This study is the first to compare the potential dependence among Lebanese smokers (cigarettes, waterpipe, and dual smokers). However, it has several limitations. Its cross-sectional design does not allow inferring causality due to temporality issues, in addition to the small subsample of dual smokers. Information bias might be present because of the difficulty in understanding some questions, and the fact survey was self-reported. Another limitation is the selection bias, as most participants were females and educated. The results are not generalizable to the entire Lebanese population due to the snowball sampling technique and the enrolled participants may not be representative of the whole Lebanese individuals. A social desirability bias is also possible as people are usually reluctant to disclose any habit considered socially undesirable or addictions that may influence their social picture [44]. Since the survey tool was available online only, this raises the possibility of missing out on a significant sample of smokers; also, the study used various social media sites that can generate different sociodemographic characteristics. Although multivariate analyses were carried out to decrease confounding, residual confounding is still possible since not all variables related to nicotine dependence could be considered. Additional studies taking into account all these limitations are necessary to confirm our findings.

## Conclusion

Our study supports the fact that heavy nicotine consumption, related to both the increased frequency and smoking duration, can increase the risk of dependence. It raises the need for strategic plans to minimize and discourage the use of nicotine products in Lebanese community settings.

## Supplementary Information

**Additional file 1: Appendix 1.** Questionnaire used in this study

## Data Availability

The data can be made available upon reasonable request to the corresponding author.

## Abbreviations

HONC: Hooked on Nicotine Checklist
LCD: Lebanese Cigarette Dependence
LWDS: Lebanese Waterpipe Dependence Scale
MANCOVA: multivariate analysis of covariance
WDLY: *Waterpipe Dependence in Lebanese Youth*
