# Supplementary material for: Association between cumulative cigarette and Waterpipe smoking and symptoms of dependence in Lebanese adults
Source: BMC Public Health. 2021 Aug 23;21:1583. doi: 10.1186/s12889-021-11626-7 (PMC8381141; doi:10.1186/s12889-021-11626-7)
Supplement: Supplementary file 1 — Additional file 1: Appendix 1. Questionnaire used in this study [file 12889_2021_11626_MOESM1_ESM.doc]

**Appendix 1. Questionnaire**

Age (years):

Gender:  Male  Female

Height: Weight:

Marital status:  Married  Single  Widowed  Divorced

Educational level:  Primary  Secondary  High School  University

Do you work?  Yes  No  Retired

Governates:  Beirut.  Mount Lebanon  North  South  Bekaa

Number of people living in the same home (including you): ______

Number of rooms in the house excluding the kitchen and bathrooms: ______

| Information about the father | Information about the mother |
| --- | --- |
| Educational level:  Primary.  Secondary.  High School  University | Educational level:  Primary.  Secondary.  High School  University |
| Does he smoke regularly?  Yes  No. If yes, what does he smoke:  Cigarettes  Waterpipe  Others: Specify | Does she smoke regularly?  Yes  No. If yes, what does she smoke:  Cigarettes  Waterpipe  Others: Specify |
| Number of cigarettes per day:  Number of waterpipes per week: | Number of cigarettes per day:  Number of waterpipes per week: |

Are you current smoker?

 Yes (even if you smoke one cigarette per day or one waterpipe in the last 30 days)

 No (if you answered No do not continue filling the survey)

**Cigarette smoking:**

At which age did you start smoking cigarettes? ________

Number of cigarettes smoked per day: ________

| Choose the answer that suits you most: | 0 | 1 | 2 | 3 |
| --- | --- | --- | --- | --- |
| Do you find it difficult to refrain from smoking where it is forbidden? | No, not at all | Yes, a little bit | Yes, somehow | Yes, too much |
| Do you smoke if you are so ill and bedridden? | No | Yes, may be | Yes, probably | Yes, sure |
| How soon after you wake up do you smoke your first cigarette? | More than 60 minutes | 31-60 minutes | 6-30 minutes | 5 minutes |
| Smoking to increase morale | No | Yes, may be | Yes, probably | Yes, sure |
| Smoking for pleasure | No | Yes, may be | Yes, probably | Yes, sure |
| Smoking to concentrate while working | No | Yes, may be | Yes, probably | Yes, sure |
| Smoking to decrease nervousness | No | Yes, may be | Yes, probably | Yes, sure |
| Smoking for conviviality | No | Yes, may be | Yes, probably | Yes, sure |
| Smoking cigarette even when alone | No | Yes, may be | Yes, probably | Yes, sure |
| Are you ready to leave your family on a holiday to go and search for cigarettes? | No | Yes, may be | Yes, probably | Yes, sure |
| Do you prefer smoking cigarettes over going to the theatre or other activities? | No | Yes, may be | Yes, probably | Yes, sure |
| Number of days you could stop smoking cigarettes | More than 7 days | 4-7 days | 2-3 days | 1 day or less |
| How much time are you ready to spend searching for cigarettes? | <30 minutes | 30 minutes -2 hours | Almost half a day | A day or more |
| How much money, in proportion to income, are you ready to pay for cigarette? | 1% of my income | 2-10 % | 10-50% | More than 50% of income |

*Hooked on Nicotine Checklist (HONC) due to cigarette smoking*

| Choose the sign x for the answer that suits you best: | | |
| --- | --- | --- |
|  | No | Yes |
| Have you ever tried to stop smoking cigarettes but could not? |  |  |
| Do you smoke / vaporize now because it is difficult to stop smoking cigarettes? |  |  |
| Have you ever felt attached to cigarettes? |  |  |
| Have you ever felt a strong craving to smoke cigarettes? |  |  |
| Have you ever felt that you really needed a cigarette? |  |  |
| In places where smoking is not permitted such as at school, do you find it difficult not to smoke cigarettes? |  |  |
| Did you find it difficult to concentrate (for example when doing homework) because you could not smoke cigarettes? |  |  |
| Did you feel that you were more agitated (excited) by your inability to smoke cigarettes? |  |  |
| Did you feel an urgent need or desire to smoke cigarettes? |  |  |
| Did you feel nervous, uncomfortable, or anxious because you could not smoke cigarettes? |  |  |

**Waterpipe smoking**

At which age did you start smoking waterpipe: _____

Number of waterpipe smoked per week: ______

*Hooked on Nicotine Checklist (HONC) due to waterpipe smoking*

| Choose the sign x for the answer that suits you best: | | |
| --- | --- | --- |
|  | No | Yes |
| Have you ever tried to stop smoking waterpipe but could not? |  |  |
| Do you smoke / vaporize now because it is difficult to stop smoking waterpipe? |  |  |
| Have you ever felt attached to waterpipes? |  |  |
| Have you ever felt a strong craving to smoke waterpipe? |  |  |
| Have you ever felt that you really needed a waterpipe? |  |  |
| In places where smoking is not permitted such as at school, do you find it difficult not to smoke waterpipes? |  |  |
| Did you find it difficult to concentrate (for example when doing homework) because you could not smoke waterpipe? |  |  |
| Did you feel that you were more agitated (excited) by your inability to smoke waterpipe? |  |  |
| Did you feel an urgent need or desire to smoke waterpipe? |  |  |
| Did you feel nervous, uncomfortable, or anxious because you could not smoke waterpipe? |  |  |

Lebanese Waterpipe Dependence Scale

| Choose the answer that suits you most: | 0 | 1 | 2 | 3 |
| --- | --- | --- | --- | --- |
| What is the number of times you could stop waterpipe for > 7 days? | It always happens | Several times | Once | None |
| What is the percent of income you would spend on waterpipe smoking? | 1% or less of your monthly income | 2-10% of your monthly income | 11-50% of your monthly income | More than 50% of your monthly income |
| What is the number of days you could spend without waterpipe? | More than 7 days | 4-7 days | 2-3 days | One day or less |
| What is the number of water pipes you usually smoke per week? | <1 waterpipe per week | 1-2 waterpipes per week | 3-6 waterpipes per week | 7 or more waterpipes per week |
| Do you smoke waterpipe to relax your nerves? | No | Yes, may be | Yes, probably | Yes, absolutely |
| Do you smoke waterpipe to improve your morale? | No | Yes, may be | Yes, probably | Yes, absolutely |
| Do you smoke waterpipe when you are seriously ill? | No | Yes, may be | Yes, probably | Yes, absolutely |
| Do you smoke waterpipe alone? | No, never | Yes, sometimes | Yes, most of the times | Yes, always |
| Are you ready not to eat in exchange for a waterpipe? | No | Yes, may be | Yes, probably | Yes, absolutely |
| Do you smoke waterpipe for pleasure? | No | Yes, may be | Yes, probably | Yes, absolutely |
| Do you smoke to please others (for conviviality) | No | Yes, may be | Yes, probably | Yes, absolutely |
